# Decreased breathing variability is associated with poorer outcome in mechanically ventilated patients

**DOI:** 10.1183/23120541.00544-2022

**Published:** 2023-05-02

**Authors:** Camille Rolland-Debord, Tymothee Poitou, Come Bureau, Isabelle Rivals, Thomas Similowski, Alexandre Demoule

**Affiliations:** 1 AP-HP, Groupe Hospitalier Universitaire APHP-Sorbonne Université, site Pitié-Salpêtrière, Service de Médecine Intensive et Réanimation (Département R3S), Paris, France; 2 Sorbonne Université, INSERM, UMRS1158 Neurophysiologie Respiratoire Expérimentale et Clinique, Paris, France; 3 Equipe de Statistique Appliquée, ESPCI Paris, PSL Research University, Paris, France; 4 AP-HP, Groupe Hospitalier Universitaire APHP-Sorbonne Université, site Pitié-Salpêtrière, Département R3S, Paris, France

## Abstract

**Rationale:**

Breathing is a cyclic activity that is variable by nature. Breathing variability is modified in mechanically ventilated patients. We aimed to evaluate whether decreased variability on the day of transition from assist-control ventilation to a partial mode of assistance was associated with a poorer outcome.

**Methods:**

This was an ancillary study of a multicentre, randomised, controlled trial comparing neurally adjusted ventilatory assist to pressure support ventilation. Flow and the electrical activity of the diaphragm (EAdi) were recorded within 48 h of switching from controlled ventilation to a partial mode of ventilatory assistance. Variability of flow and EAdi-related variables were quantified by the coefficient of variation, the amplitude ratio of the spectrum's first harmonic to its zero-frequency component (H1/DC) and two surrogates of complexity.

**Main results:**

98 patients ventilated for a median duration of 5 days were included. H1/DC of inspiratory flow and EAdi were lower in survivors than in nonsurvivors, suggesting a higher breathing variability in this population (for flow, 37% *versus* 45%, p=0.041; for EAdi, 42% *versus* 52%, p=0.002). By multivariate analysis, H1/DC of inspiratory EAdi was independently associated with day-28 mortality (OR 1.10, p=0.002). H1/DC of inspiratory EAdi was lower in patients with a duration of mechanical ventilation <8 days (41% *versus* 45%, p=0.022). Noise limit and the largest Lyapunov exponent suggested a lower complexity in patients with a duration of mechanical ventilation <8 days.

**Conclusion:**

Higher breathing variability and lower complexity are associated with higher survival and lower duration of mechanical ventilation.

## Abstract 2

**In mechanically ventilated patients studied at the transition from assist-control ventilation to a partial mode of assistance, higher breath-by-breath variability and spectral variability were associated with better outcomes** https://bit.ly/3KMKJ3v

## Introduction

Breathing is a cyclic activity that is not monotonous, but exhibits natural variability [1, 2]. In normal human subjects, ventilation shows breath-by-breath variability in descriptors of breathing pattern such as respiratory rate and tidal volume [2]. Breathing variability can also be characterised by the spectral analysis of the flow signal [3, 4]. Finally, breathing activity is nonlinear in nature and exhibits chaos-like mathematical complexity [5, 6].

In the intensive care unit (ICU), decreased breath-by-breath variability in mechanically ventilated patients is associated with weaning failure [3, 7], and one study showed that alterations of respiratory rate spectral analysis are associated with increased mortality [5]. However, in this latter study, variability was quantified globally, from the initiation of mechanical ventilation to extubation, which precluded the use of variability as a prognostic index at a given time point of ICU stay. In addition, in these studies, breathing variability was restricted to downstream variables such as airway flow and tidal volume, while the upstream variability of the central inspiratory activity was ignored. It is worth noting that the electromyographic activity of the diaphragm depends directly on the central inspiratory activity [8]. Finally, variability was generally assessed using one single tool of analysis. A recent review describes breathing variability in anaesthesia and critical care, suggesting that variability of respiration is not yet fully understood and that the respiratory system should be measured as a whole rather than a single parameter [9].

Here, we performed an ancillary study of a multicentre, randomised, controlled trial. We described and quantified variability by an array of descriptors, including breath-by-breath variability, spectral analysis and mathematical complexity. This quantification was achieved at the transition between assist-control ventilation and ventilation with a partial mode; in other words, as soon as patients could sustain pressure support ventilation (PSV). We chose this time point because this is the first moment during mechanical ventilation that the brain resumes its control over ventilatory activity and therefore the first moment that the natural variability of the respiratory system can be evaluated, since this natural variability was previously occulted by control ventilation [10]. We hypothesised that a low variability at the time of the switch to partial ventilatory mode could predict a poorer outcome.

## Methods

This is a pre-planned ancillary study of a multicentre, randomised, controlled trial that aimed to compare neurally adjusted ventilatory assist (NAVA) to PSV in mechanically ventilated patients in 11 ICU departments in France [11]. The study protocol was approved for all centres by the Comité de Protection des Personnes Ile de France 8 (no. 2010-A00424–35), according to French law. A detailed description of the study design and data from this cohort has been published previously [11, 12].

### Patients

Patients receiving mechanical ventilation for >24 h for acute respiratory failure of respiratory cause were eligible when they met the following criteria: ability to sustain PSV for ≥30 min with a total level of inspiratory pressure <30 cmH_2_O, estimated remaining duration of mechanical ventilation >48 h, level of sedation (Ramsay scale) ≤4, fraction of inspired oxygen ≤50% with a positive end-expiratory pressure ≤8 cmH_2_O and absence of administration of high-dose vasopressor therapy. Exclusion criteria were age <18 years, known pregnancy, participation in another trial within the 30 days preceding satisfaction of the eligibility criteria, contraindication of the implementation of the oesophageal tube and decision to withhold life-sustaining treatment.

### Patient management

After inclusion, patients were connected to a Servo-i ventilator (Maquet Critical Care, Sweden) equipped with NAVA mode. An extensive description of patient management is provided in the supplementary methods.

### Data collection

Airway pressure, airway flow and the electrical activity of the diaphragm (EAdi) were recorded 12, 24, 36 and 48 h after inclusion. They were acquired for 20 min at 100 Hz from the ventilator connected to a computer using commercially available software (Servo-i RCR, version 3.6.2; Maquet Critical Care). Outcome data included mortality 28 days after inclusion, duration of mechanical ventilation and ventilator-free days (VFDs) 28 days after inclusion.

### Data analysis

**FIGURE 1:**
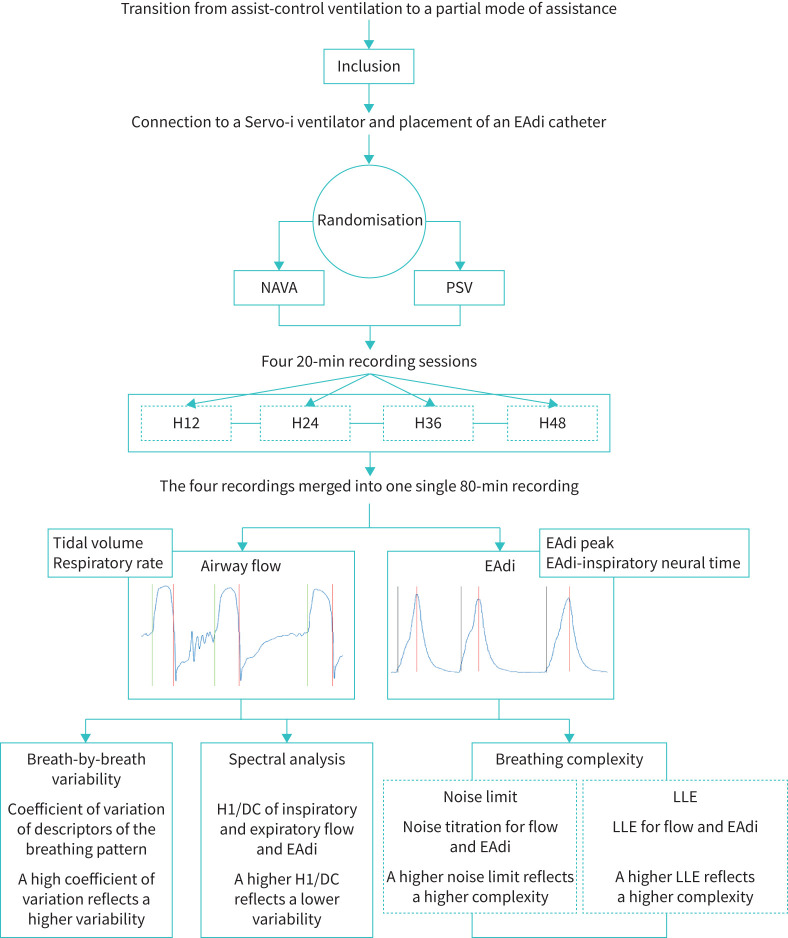
Experimental design. EAdi: electrical activity of the diaphragm; NAVA: neurally adjusted ventilatory assist; PSV: pressure support ventilation; H1/DC: amplitude ratio of the first harmonic peak (H1) to that of zero frequency (also termed DC component); LLE: largest Lyapunov exponent.

For each patient, the four 20-min recordings (12, 24, 36 and 48 h after inclusion) were merged into one single 80-min recording session on which analyses were performed (figure 1). An extensive description of signal processing and data analysis is provided in the supplementary methods.

Breath-by-breath variability of flow-derived and EAdi-derived breathing pattern variables was assessed by the coefficient of variation (standard deviation divided by the mean; the higher the coefficient of variation, the higher the variability). Flow-derived breathing pattern variables included tidal volume and respiratory rate. For EAdi, peak EAdi (EAdi-peak) and EAdi-inspiratory neural time were determined.

Spectral-derived variability was assessed using the amplitude ratio of the spectrum's first harmonic (H1) to its zero-frequency or DC component (H1/DC) according to the method described by Gutierrez *et al*. [4] (the higher the H1/DC, the lower the variability).

Breathing complexity was assessed by the noise limit and largest Lyapunov exponent [13]. A noise limit above zero means nonlinearity and a certain degree of complexity [10, 14, 15]. Sensitivity to initial conditions is how perturbations occurring in the past affect the future behaviour of the system and is another characteristic of how a complex system is unpredictable. This was estimated for flow and EAdi using the largest Lyapunov exponent [13].

### Statistics

As this is an ancillary study, no sample size could be calculated to detect a difference. The sample size was determined by the parent study [11]. Statistical analysis was performed using GraphPad (GraphPad Software, San Diego, CA, USA) and R (The R Foundation, Vienna, Austria). Continuous data were reported as median (interquartile range) and categorical data as number of events (percentage). Continuous variables (*i.e.* duration of mechanical ventilation and number of 28-day VFDs) were dichotomised according to their median value in the population.

Differences between groups were assessed with the Mann–Whitney test for continuous variables and with the Chi-squared test for categorical variables. Each potential risk factor for death was first evaluated in a univariate model. Then, a multivariate logistic regression analysis was performed. The multivariate model was built with variables that yielded p-values of <0.2 on univariate analysis. The adjusted odds ratios of variables present in the final model are presented with a 95% confidence interval. Finally, correlation between duration of mechanical ventilation, 28-day VFDs and descriptors of breathing variability were evaluated using Spearman's rank correlation coefficient.

## Results

### Study population

128 patients were included in the parent study: 62 in the NAVA group and 66 in the PSV group. For technical reasons, flow, pressure and EAdi analysis failed in 14 patients of the NAVA group and 16 patients of the PSV group. Subsequently, data on breathing variability were available for 98 patients: 48 in the NAVA group and 50 in the PSV group. The main characteristics of the patients are displayed in table 1.

**TABLE 1:** Baseline characteristics of the patients

**Patients**	98
**Male**	65 (66)
**Age, years**	68 (60–77)
**SAPS II**	44 (34–59)
**Charlson score**	3 (2–5)
**ATICE**	16 (11–19)
**Duration of controlled or assist-control ventilation prior to switch to partial mode, days**	5 (3–9)
**Duration of mechanical ventilation, days**	8 (4–13)
**Ventilator-free days 28 days after inclusion**	23 (10–25)
**Mortality within 28 days**	19 (19)
**Cause of ARF**	
*De novo* ARF	57 (58)
Post-operative ARF	19 (19)
Acute-on-chronic ARF	17 (18)
Acute cardiogenic pulmonary oedema	5 (5)
**Ventilator measurements, at inclusion**	
PEEP, cmH_2_O	6 (5–8)
PSV level,^#^ cmH_2_O	12 (10–13)
NAVA level,^¶^ cmH_2_O·μV^−1^	1.6 (1.2–2.3)
**Breathing pattern at inclusion**	
Tidal volume, mL	450 (400–525)
Respiratory rate, min^−1^	24 (20–29)
Minute ventilation, L·min^−1^	11 (9–13)
**Blood gases**	
*P*_aO_2__/*F*_IO_2__ at inclusion, mmHg	240 (193–286)
*P*_aCO_2__ at inclusion, mmHg	40 (35–48)

Data are presented as n, n (%) or median (interquartile range). SAPS: simplified acute physiology score; ATICE: adaptation to the intensive care environment; ARF: acute respiratory failure; PEEP: positive end-expiratory pressure; PSV: pressure support ventilation; NAVA: neurally adjusted ventilatory assist; *P*_aO_2__: arterial oxygen tension; *F*_IO_2__: inspiratory oxygen fraction; *P*_aCO_2__: arterial carbon dioxide tension. ^#^: reported for the 50 patients mechanically ventilated with the PSV mode; ^¶^: reported for the 48 patients mechanically ventilated with the NAVA mode.

Supplementary table SDC1 compares the descriptors of breathing variability between patients assigned to the NAVA group and those assigned to the PSV group in the mother trial. The coefficient of variation of the tidal volume and the largest Lyapunov exponent for flow were higher in patients assigned to the NAVA group as compared to those assigned to the PSV group.

### Association between breathing variability and 28-day mortality

Mortality within 28 days was 19% (n=19). Table 2 shows the descriptors of breathing variability associated with 28-day mortality by univariate analysis. Among descriptors of breathing variability, two differed between survivors and nonsurvivors. H1/DC of inspiratory flow and H1/DC of inspiratory EAdi were lower in survivors, suggesting a higher variability in this population. By multivariate analysis, H1/DC of inspiratory EAdi was the only factor independently associated with 28-day mortality (OR 1.10, 95% CI 1.04–1.17; p=0.002).

**TABLE 2:** Association between descriptors of breathing variability and 28-day mortality

	**Survivors**	**Nonsurvivors**	**p-value**
**Patients**	79	19	
**Baseline characteristics**			
Male	51 (65)	14 (74)	0.408
Age, years	66 (58–76)	75 (69–79)	0.015
SAPS II	42 (33–54)	48 (42–67)	0.016
Charlson score	4 (2–5)	3 (2–4)	0.174
ATICE	16 (11–19)	14 (10–19)	0.304
Duration of mechanical ventilation prior to inclusion, days	5 (2–9)	6 (3–9)	0.751
**Cause of ARF**			
*De novo* ARF	45 (57)	12 (63)	0.596
Post-operative ARF	16 (20)	3 (16)	1
Acute-on-chronic ARF	14 (18)	3 (16)	1
Acute cardiogenic pulmonary oedema	4 (5)	1 (5)	1
**Ventilator measurements, at inclusion**			
PEEP, cmH_2_O	6 (5–8)	8 (6–8)	0.031
PSV level,^#^ cmH_2_O	12 (10–12)	14 (8–17)	0.611
NAVA level,^¶^ cmH_2_O·μV^−1^	1.6 (1.2–2.3)	1.9 (1.4–2.3)	0.481
**Breathing pattern, at inclusion**			
Tidal volume, mL	440 (400–520)	445 (381–541)	0.932
Respiratory rate, min^−1^	24 (20–29)	26 (20–29)	0.635
Minute ventilation, L·min^−1^	11 (9–13)	12 (10–14)	0.285
**Blood gases**			
*P*_aO_2__/*F*_IO_2__ at inclusion, mmHg	240 (195–292)	226 (186–264)	0.355
*P*_aCO_2__ at inclusion, mmHg	39 (34–45)	41 (35–51)	0.442
**Descriptors of breathing variability**			
Coefficient of variation			
Tidal volume, %	22 (16–34)	21 (14–25)	0.150
Respiratory rate, %	23 (18–30)	22 (14–26)	0.263
EAdi-peak, %	34 (27–46)	35 (24–46)	0.754
EAdi-inspiratory neural time, %	31 (27–38)	31 (23–52)	0.864
H1/DC			
Inspiratory flow, %	37 (31–44)	45 (33–52)	0.041
Inspiratory EAdi, %	42 (34–48)	52 (41–58)	0.002
Expiratory flow, %	23 (18–30)	24 (19–31)	0.614
Expiratory EAdi, %	30 (24–35)	34 (29–36)	0.091
Complexity			
Noise limit flow, %	46 (33–65)	50 (38–82)	0.241
Noise limit EAdi, %	45 (34–66)	51 (37–83)	0.384
LLE flow, bit·iteration^−1^	2.15 (1.61–2.65)	2.32 (1.84–2.54)	0.583
LLE EAdi, bit·iteration^−1^	0.21 (0.11–0.34)	0.19 (0.11–0.49)	0.793

Data are presented as n, n (%) or median (interquartile range), unless otherwise stated. SAPS: simplified acute physiology score; ATICE: adaptation to the intensive care environment; ARF: acute respiratory failure; PEEP: positive end-expiratory pressure; PSV: pressure support ventilation; NAVA: neurally adjusted ventilatory assist; *P*_aO_2__: arterial oxygen tension; *F*_IO_2__: inspiratory oxygen fraction; *P*_aCO_2__: arterial carbon dioxide tension; EAdi: electrical activity of the diaphragm; H1/DC: amplitude ratio of the first harmonic peak (H1) to that of zero frequency (also termed DC component); LLE: largest Lyapunov exponent. ^#^: reported for the 50 patients mechanically ventilated with the PSV mode; ^¶^: reported for the 48 patients mechanically ventilated with the NAVA mode.

### Association between breathing variability and duration of mechanical ventilation

Duration of mechanical ventilation was 8 (4–13) days. Table 3 shows the descriptors of breathing variability associated with duration of mechanical ventilation. Among descriptors of breathing variability, three differed between patients with a duration of mechanical ventilation <8 days and those with a duration of mechanical ventilation ≥8 days. H1/DC of inspiratory EAdi was lower in patients with a duration of mechanical ventilation <8 days, and there was a significant, but poor, correlation between H1/DC of inspiratory flow and EAdi and duration of mechanical ventilation (supplementary figure SDC1). This suggested a higher breathing variability in patients with a shorter duration of mechanical ventilation. Noise limit for respiratory flow and EAdi was higher in patients with a longer duration of mechanical ventilation, and there was a positive correlation between noise limit for respiratory flow and EAdi and duration of mechanical ventilation. This suggested an association between a higher complexity and a longer duration of mechanical ventilation (supplementary figure SDC1, supplementary table SDC2).

**TABLE 3:** Association between descriptors of breathing variability and duration of mechanical ventilation

	**Duration of mechanical ventilation** **<8** **days**	**Duration of mechanical ventilation** ≥**8** **days**	**p-value**
**Patients**	49	49	
**Baseline characteristics**			
Male	32 (65)	33 (67)	0.831
Age, years	68 (57–77)	66 (61–77)	0.507
SAPS II	37 (31–49)	44 (39–44)	0.021
Charlson score	5 (3–6)	5 (4–6)	0.466
ATICE	16 (11–19)	15 (11–18)	0.549
Duration of mechanical ventilation prior to inclusion, days	5 (2–8)	7 (4–10)	0.259
**Cause of ARF**			
*De novo* ARF	28 (59)	26 (54)	0.684
Post-operative ARF	11 (21)	9 (18)	0.616
Acute-on-chronic ARF	6 (12)	10 (20)	0.274
Acute cardiogenic pulmonary oedema	4 (8)	4 (8)	1
**Ventilator measurements, at inclusion**			
PEEP, cmH_2_O	6 (5–8)	7 (5–8)	0.384
PSV level,^#^ cmH_2_O	12 (10–12)	12 (10–16)	0.182
NAVA level,^¶^ cmH_2_O·μV^−1^	1.6 (1.2–2.0)	1.9 (1.2–2.3)	0.403
**Breathing pattern**			
Tidal volume, mL	440 (400–511)	445 (385–547)	0.882
Respiratory rate, min^−1^	23 (18–30)	24 (21–28)	0.918
Minute ventilation, L·min^−1^	11 (9–13)	11 (9–13)	0.926
**Blood gases**			
*P*_aO_2__/*F*_IO_2__ at inclusion, mmHg	251 (207–317)	213 (185–266)	0.021
Mean *P*_aO_2__/*F*_IO_2___,_ mmHg	228 (167–314)	214 (174–266)	0.347
*P*_aCO_2__ at inclusion, mmHg	39 (34–44)	41 (35–48)	0.204
**Descriptors of breathing variability**			
Coefficient of variation			
Tidal volume, %	24 (16–34)	19 (15–30)	0.113
Respiratory rate, %	23 (19–30)	22 (17–29)	0.378
EAdi-peak, %	35 (27–47)	34 (27–46)	0.864
EAdi-inspiratory neural time, %	31 (27–39)	31 (26–42)	0.963
H1/DC			
Inspiratory flow, %	37 (30–42)	39 (34–50)	0.071
Inspiratory EAdi, %	41 (31–48)	45 (39–52)	0.022
Expiratory flow, %	23 (17–31)	23 (20–30)	0.684
Expiratory EAdi, %	30 (24–35)	31 (25–36)	0.318
Complexity			
Noise limit, flow, %	42 (31–61)	56 (39–68)	0.006
Noise limit, EAdi, %	41 (31–60)	56 (41–70)	0.008
LLE, flow, bit.iteration^−1^	2.1 (1.6–2.6)	2.3 (1.6–2.7)	0.453
LLE, EAdi, bit.iteration^−1^	0.21 (0.11–0.38)	0.21 (0.14–0.33)	0.774

Data are presented as n, n (%) or median (interquartile range), unless otherwise stated. SAPS: simplified acute physiology score; ATICE: adaptation to the intensive care environment; ARF: acute respiratory failure; PEEP: positive end-expiratory pressure; PSV: pressure support ventilation; NAVA: neurally adjusted ventilatory assist; *P*_aO_2__: arterial oxygen tension; *F*_IO_2__: inspiratory oxygen fraction; *P*_aCO_2__: arterial carbon dioxide tension; EAdi: electrical activity of the diaphragm; H1/DC: amplitude ratio of the first harmonic peak (H1) to that of zero frequency (also termed DC component); LLE: largest Lyapunov exponent. ^#^: reported for the 50 patients mechanically ventilated with the PSV mode; ^¶^: reported for the 48 patients mechanically ventilated with the NAVA mode.

### Association between breathing variability and 28-day VFDs

**FIGURE 2:**
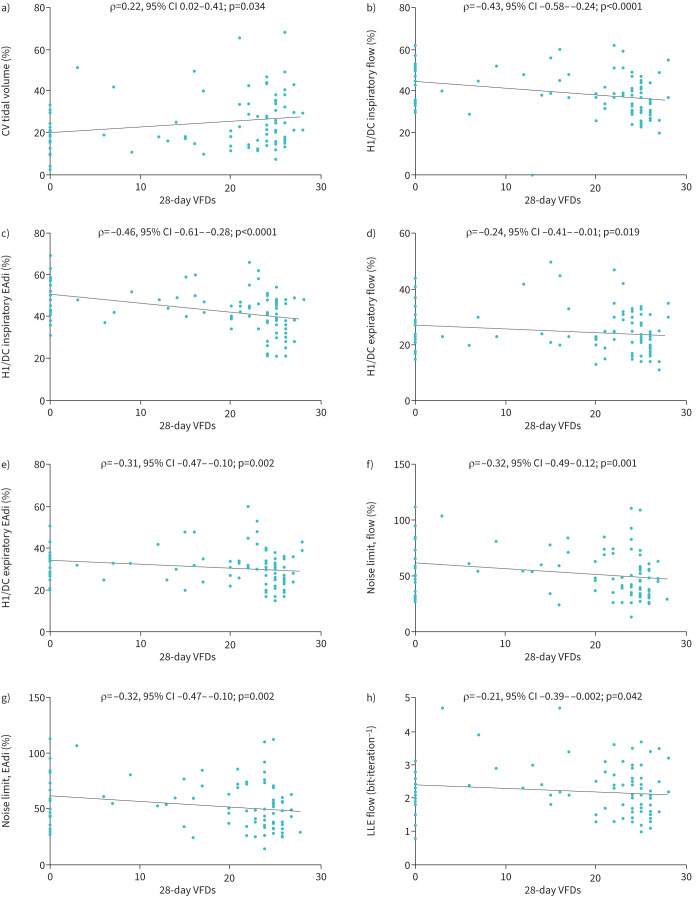
Correlation between 28-day ventilator-free days (VFDs) and descriptors of breathing variability. a) Coefficient of variation (CV) of tidal volume; b) amplitude ratio of the first harmonic peak (H1) to that of zero frequency (also termed DC component) inspiratory flow; c) H1/DC inspiratory electrical activity of the diaphragm (EAdi); d) H1/DC expiratory flow; e) H1/DC expiratory EAdi; f) noise limit flow; g) noise limit EAdi; h) largest Lyapunov exponent (LLE) flow evaluated using Spearman's rank correlation coefficient..

Ventilator-free duration 28 days after inclusion was 23 (10–25) days. Table 4 shows the association between descriptors of breathing variability and 28-day VFDs. Among descriptors of breathing variability, eight differed between patients with 28-day VFDs <23 days and those with 28-day VFDs ≥23 days. Among patients with 28-day VFDs ≥23 days, the coefficient of variation of the tidal volume was higher and the inspiratory and expiratory H1/DC for EAdi and flow were lower, suggesting an association between a higher variability and an increased number of 28-day VFDs. Correlations between these variables and 28-day VFDs conveyed the same message. Among patients with 28-day VFDs <23 days, the noise limit of flow and EAdi and largest Lyapunov exponent of flow were higher, with a correlation between these variables and 28-day VFDs. These results suggested that a higher complexity was associated with fewer 28-day VFDs (figure 2 and supplementary table SDC3).

**TABLE 4:** Association between descriptors of breathing variability and 28-day ventilator-free days (VFDs)

	**28-day VFDs** **<23** **days**	**28-day VFDs** ≥**23** **days**	**p-value**
**Patients**	49	49	
**Baseline characteristics**			
Male	36 (74)	29 (59)	0.558
Age, years	72 (63–78)	64 (57–74)	0.031
SAPS II	48 (40–63)	35 (26–45)	<0.0001
Charlson score	5 (4–7)	5 (4–6)	0.285
ATICE	15 (11–19)	16 (11–18)	0.194
Duration of mechanical ventilation prior to inclusion, days	6 (3–9)	5(1–14)	0.897
**Cause of acute respiratory failure**			
*De novo* ARF	31 (64)	23 (47)	0.154
Post-operative ARF	8 (16)	12 (25)	0.452
Acute-on-chronic ARF	7 (14)	9 (18)	0.785
Acute cardiogenic pulmonary oedema	3 (6)	5 (10)	0.714
**Ventilator measurements, at inclusion**			
PEEP, cmH_2_O	6 (5–8)	7 (5–8)	0.850
PSV level,^#^ cmH_2_O	12 (11–16)	11(10–12)	0.040
NAVA level,^¶^ cmH_2_O·μV^−1^	1.8 (1.2–2.3)	1.6 (1.1–2)	0.452
**Breathing pattern**			
Tidal volume, mL	440 (400–547)	450 (398–518)	0.954
Respiratory rate, min^−1^	24 (22–29)	23 (18–27)	0.094
Minute ventilation, L·min^−1^	11 (10–14)	10 (8–12)	0.041
**Blood gases**			
*P*_aO_2__/*F*_iO_2__ at inclusion, mmHg	225 (184–266)	249 (202–298)	0.044
Mean *P*_aO_2__/*F*_iO_2___,_ mmHg	214 (176–253)	231 (164–316)	0.388
*P*_aCO_2__ at inclusion, mmHg	40 (35–45)	40 (34–48)	0.951
Descriptors of breathing variability			
Coefficient of variation			
Tidal volume, %	19 (14–28)	25 (17–36)	0.023
Respiratory rate, %	22 (16–26)	24 (19–30)	0.071
EAdi-peak, %	32 (26–45)	37 (30–49)	0.087
EAdi-inspiratory neural time, %	30 (23–42)	33 (28–39)	0.327
H1/DC			
Inspiratory flow, %	42 (37–52)	34 (29–41)	<0.0001
Inspiratory EAdi, %	47 (41–58)	38 (30–46)	<0.0001
Expiratory flow, %	24 (21–32)	22 (16–28)	0.007
Expiratory EAdi, %	33 (28–37)	26 (23–33)	0.0002
Complexity			
Noise limit, flow, %	52 (40–73)	41 (31–60)	0.012
Noise limit, EAdi, %	53 (40–73)	42 (31–60)	0.013
LLE, flow, bit.iteration^−1^	2.32 (1.85–2.78)	2.00 (1.54–2.51)	0.028
LLE, EAdi, bit.iteration^−1^	0.22 (0.12–0.39)	0.20 (0.11–0.31)	0.323

Data are presented as n, n (%) or median (interquartile range), unless otherwise stated. SAPS: simplified acute physiology score; ATICE: adaptation to the intensive care environment; ARF: acute respiratory failure; PEEP: positive end-expiratory pressure; PSV: pressure support ventilation; NAVA: neurally adjusted ventilatory assist; *P*_aO_2__: arterial oxygen tension; *F*_IO_2__: inspiratory oxygen fraction; *P*_aCO_2__: arterial carbon dioxide tension; EAdi: electrical activity of the diaphragm; H1/DC: amplitude ratio of the first harmonic peak (H1) to that of zero frequency (also termed DC component); LLE: largest Lyapunov exponent. ^#^: reported for the 50 patients mechanically ventilated with the PSV mode; ^¶^: reported for the 48 patients mechanically ventilated with the NAVA mode.

## Discussion

The main findings in our cohort of 98 mechanically ventilated patients studied at the early phase of weaning can be summarised as follows: 1) higher breath-by-breath variability as assessed by the coefficient of variation, and higher spectral variability as assessed by the H1/DC ratio, were associated with a lower mortality and a lower duration of mechanical ventilation, resulting in increases in VFDs; 2) higher complexity as assessed by noise limit and the largest Lyapunov exponent was associated with a longer duration of mechanical ventilation and fewer VFDs.

To our knowledge, this is the first study to evaluate in a large population the prognostic impact of reduced variability and complexity in the ICU at one given time point (*i.e.* the transition between assist-control ventilation and a partial mode of assistance such as PSV or NAVA) and with several flow- and EAdi-derived indices to quantify breath-by-breath variability, spectral-derived variability and complexity. Previous studies on this topic used only one of these approaches and did not integrate EAdi into their analyses.

### Relationship between variability and outcome

A major result was that higher breath-by-breath and spectral variability were associated with a better outcome. This result is in line with previous reports showing that a higher breath-by-breath variability is associated with a higher weaning success rate [7, 16]. A body of literature suggests an inverse relationship between breathing variability and respiratory system loading [17–19]. In mechanical ventilation patients, unloading the respiratory system is associated with higher respiratory variability [20, 21]. These results suggest that respiratory variability parallels the load–capacity balance of the respiratory system. A high variability may witness a large respiratory reserve [7], and subsequently a higher likeliness to be weaned with, in turn, a shorter duration of mechanical ventilation [22]. Regarding the association between higher spectral variability as assessed by the H1/DC ratio and lower mortality, our findings confirm the previous report from Gutierrez *et al*. [3].

### Relationship between complexity and outcome

Greater complexity was associated with a longer duration of mechanical ventilation and fewer VFDs. Ventilatory flow is not periodic [5], but exhibits complexity, with this term implying irregularity, sensitivity to initial conditions and unpredictability. In other words, this is the amount of “surprise” or “new information” introduced into an otherwise predictable system, *i.e.* the degree of disorder or randomness in the data [9]. In animals and humans, ventilator complexity has been characterised by various mathematical approaches such as correlation dimension, approximate entropy, Lyapunov exponents and noise limit, which investigates the chaotic nature of ventilator flow [23].

Few studies have evaluated the relationship between complexity and outcome in ICU patients. These studies are in line with our results. The study by El-Khatib *et al*. [24] showed that the breathing pattern measured by Kolmogorov entropy and respiratory flow–volume phase space dimension during mechanical ventilation was more complex and chaotic in patients who failed weaning than in those who succeeded. The study by Engoren *et al*. [25] found similar results. Patients who failed weaning showed increased irregularity in the biosignal analysis of approximate tidal volume entropy, which, according to the authors, reflected enhanced external inputs to the respiratory control centre. They suggested that increased regularity in the weaning success group indicated a better adaptive mechanism of an autonomous system. Finally, Park *et al*. [26] found that the electrocardiogram and photoplethysmography exhibited more complex and chaotic behaviour in patients who failed weaning.

### Clinical implications and perspectives

Our results suggest that breathing variability measured at a given time point, the transition between assist-control ventilation and a partial mode of assistance, could be used as a predictor of duration of mechanical ventilation and even survival. This may help in deciding important therapeutic options such as hastening the weaning process or, conversely, performing a tracheostomy.

It is worth noting that analyses derived from the upstream EAdi signal did not provide much more information than analyses derived from the downstream flow signal, which will simplify the assessment of variability in daily practice, since recording the respiratory flow signal is much easier than recording the EAdi. This result was quite surprising, since EAdi is a closer surrogate of the activity and hence variability of the central respiratory pattern generators located in the brainstem [8]. It suggests that the prognostic value of breathing variability results not only from the central respiratory pattern generator from where it originates [23], but also from the way the respiratory system alters this neural variability, which relates to the load-capacity relationship of the respiratory system [17, 18, 27].

Because greater variability is associated with a better outcome, one can hypothesise that restoring variability could improve the outcome. A body of literature suggests that, during mechanical ventilation, greater variability may be associated with a more protective ventilation. In mechanically ventilated animals, decreased variability of tidal volume is associated with altered lung mechanics and increased lung damage [28], and the restoration of a certain level of variability [29–31] improves respiratory system compliance and the secretion of surfactant, decreases histological lung damage and lung inflammation and improves gas exchange [28–32]. Restoring variability could involve the restoration of the intrinsic variability of the respiratory system with a proportional mode of ventilation such as NAVA or proportional assist ventilation [8, 33]. Previous studies have shown that breath-by-breath variability is higher with these modes than with pressure support ventilation [8, 33]. This could involve the introduction of a certain level of extrinsic variability with modes of mechanical ventilation such as variable or “noisy” pressure support ventilation [34, 35]. In mechanically ventilated patients, this mode is associated with improved gas exchange [22].

From a clinical perspective, our results pave the way for future studies evaluating how breathing variability could be used to improve the management of mechanical ventilation. For instance, combined with other anamnestic or clinical data, breathing variability may help to determine the outcome of a patients transitioning from assist-control ventilation to pressure support.

In the era of artificial intelligence and personalised medicine, our results could be later used as a predictive algorithm for weaning success or failure and to adjust the promptness of transition from controlled and assist-control ventilation to a partial mode of assistance. In addition, mechanically ventilated patients at high risk of mortality will be more easily identified.

### Strength and limitations of the study

The strengths of this study include the unselected character of our population of ICU patients, which is quite representative of a standard ICU population given its characteristics, severity and outcome. The multicentre design, involving 11 ICUs, enhances the generalisability of our findings. Finally, all the patients were studied at a given and comparable time point. This study presents a number of limitations. First, the sample size was not calculated *a priori* because it was a secondary analysis. Second, the recordings could not be analysed in some patients, which reduced the sample size and in turn decreased the power of the study. Third, some of the indices we used required long and complex mathematical processing, which limits the immediate transposition of our results. Fourthly, aggregating measurements made over 48 h could “dilute” the moment when the brain recovers its aptitude to generate variability. However, limiting the analysis to the first recording would have limited the quality of analyses due to the short duration (20 min) of the recording. Finally, our study suggests how to monitor the transition from assist-control ventilation to a partial mode of assistance in a large but heterogeneous population and confounders as disease severity, comorbidities, baseline diagnostics may have impacted the results. Further studies are therefore needed to determine in more balanced groups the impact of our measurements. In this preliminary, and by no means exhaustive study on the use of an array of variability and complexity descriptors, it would be nice to further compared the analysed parameters between the different weaning groups (*i.e.* short, difficult and prolonged weaning) keeping only the analysis of the significant parameters identified in this work.

### Conclusion

In mechanically ventilated patients studied at the transition from assist-control ventilation to a partial mode of assistance, higher breath-by-breath variability and spectral variability were associated with better outcomes. These results pave the way for future studies that will evaluate more precisely the accuracy of these indices, which time point is the more reliable to gather them, and if repeated measures could improve this accuracy. Obviously, these studies will require the development of automated tools. In addition, these results support trials that would evaluate the prognostic impact of strategies aiming at restoring a more physiological level of variability in mechanically ventilated patients, although this physiological level is as yet unknown [2, 36].

## Supplementary material

10.1183/23120541.00544-2022.Supp1 **Please note:** supplementary material is not edited by the Editorial Office, and is uploaded as it has been supplied by the author.
Supplementary material 00544-2022.supplement

## References

[1] Priban IP. An analysis of some short-term patterns of breathing in man at rest. J Physiol 1963; 166: 425–434. doi:10.1113/jphysiol.1963.sp007114 13986111 PMC1359344

[2] Tobin MJ, Mador MJ, Guenther SM, et al. Variability of resting respiratory drive and timing in healthy subjects. J Appl Physiol 1988; 65: 309–317. doi:10.1152/jappl.1988.65.1.309 3403474

[3] Gutierrez G, Das A, Ballarino G, et al. Decreased respiratory rate variability during mechanical ventilation is associated with increased mortality. Intensive Care Med 2013; 39: 1359–1367. doi:10.1007/s00134-013-2937-5 23743521

[4] Gutierrez G, Ballarino GJ, Turkan H, et al. Automatic detection of patient-ventilator asynchrony by spectral analysis of airway flow. Crit Care 2011; 15: R167. doi:10.1186/cc10309 21749683 PMC3387605

[5] Modarreszadeh M, Bruce EN, Gothe B. Nonrandom variability in respiratory cycle parameters of humans during stage 2 sleep. J Appl Physiol 1990; 69: 630–639. doi:10.1152/jappl.1990.69.2.630 2228875

[6] Benchetrit G, Bertrand F. A short-term memory in the respiratory centres: statistical analysis. Respir Physiol 1975; 23: 147–158. doi:10.1016/0034-5687(75)90056-0 1144937

[7] Wysocki M, Cracco C, Teixeira A, et al. Reduced breathing variability as a predictor of unsuccessful patient separation from mechanical ventilation. Crit Care Med 2006; 34: 2076–2083. doi:10.1097/01.CCM.0000227175.83575.E9 16755257

[8] Schmidt M, Demoule A, Cracco C, et al. Neurally adjusted ventilatory assist increases respiratory variability and complexity in acute respiratory failure. Anesthesiology 2010; 112: 670–681. doi:10.1097/ALN.0b013e3181cea375 20179505

[9] van den Bosch OFC, Alvarez-Jimenez R, de Grooth HJ, et al. Breathing variability – implications for anaesthesiology and intensive care. Crit Care 2021; 25: 280. doi:10.1186/s13054-021-03716-0 34353348 PMC8339683

[10] Mangin L, Fiamma MN, Straus C, et al. Source of human ventilatory chaos: lessons from switching controlled mechanical ventilation to inspiratory pressure support in critically ill patients. Respir Physiol Neurobiol 2008; 161: 189–196. doi:10.1016/j.resp.2008.02.006 18387347

[11] Demoule A, Clavel M, Rolland-Debord C, et al. Neurally adjusted ventilatory assist as an alternative to pressure support ventilation in adults: a French multicentre randomized trial. Intensive Care Med 2016; 42: 1723–1732. doi:10.1007/s00134-016-4447-8 27686347

[12] Rolland-Debord C, Bureau C, Poitou T, et al. Prevalence and prognosis impact of patient–ventilator asynchrony in early phase of weaning according to two detection methods. Anesthesiology 2017; 127: 989–997. doi:10.1097/ALN.0000000000001886 28914623

[13] Briggs K. An improved method for estimating Liapunov exponents of chaotic time series. Phys Lett A 1990; 151: 27–32. doi:10.1016/0375-9601(90)90841-B

[14] Schmidt M, Kindler F, Cecchini J, et al. Neurally adjusted ventilatory assist and proportional assist ventilation both improve patient–ventilator interaction. Crit Care 2015; 19: 56. doi:10.1186/s13054-015-0763-6 25879592 PMC4355459

[15] Roulin E, Freitas US, Letellier C. Working conditions for safe detection of nonlinearity and noise titration. Phys Rev E Stat Nonlin Soft Matter Phys 2011; 83: 046225. doi:10.1103/PhysRevE.83.046225 21599288

[16] Bien MY, Hseu SS, Yien HW, et al. Breathing pattern variability: a weaning predictor in postoperative patients recovering from systemic inflammatory response syndrome. Intensive Care Med 2004; 30: 241–247. doi:10.1007/s00134-003-2073-8 14647889

[17] Brack T, Jubran A, Tobin MJ. Effect of elastic loading on variational activity of breathing. Am J Respir Crit Care Med 1997; 155: 1341–1348. doi:10.1164/ajrccm.155.4.9105077 9105077

[18] Brack T, Jubran A, Tobin MJ. Effect of resistive loading on variational activity of breathing. Am J Respir Crit Care Med 1998; 157: 1756–1763. doi:10.1164/ajrccm.157.6.9704114 9620902

[19] Jubran A, Grant BJ, Tobin MJ. Effect of hyperoxic hypercapnia on variational activity of breathing. Am J Respir Crit Care Med 1997; 156: 1129–1139. doi:10.1164/ajrccm.156.4.97-01080 9351612

[20] Schmidt M, Demoule A, Polito A, et al. Dyspnea in mechanically ventilated critically ill patients. Crit Care Med 2011; 39: 2059–2065. doi:10.1097/CCM.0b013e31821e8779 21572329

[21] Schmidt M, Boutmy-Deslandes E, Perbet S, et al. Differential perceptions of noninvasive ventilation in intensive care among medical caregivers, patients, and their relatives: a multicenter prospective study – the PARVENIR study. Anesthesiology 2016; 124: 1347–1359. doi:10.1097/ALN.0000000000001124 27035854

[22] Spieth PM, Güldner A, Beda A, et al. Comparative effects of proportional assist and variable pressure support ventilation on lung function and damage in experimental lung injury. Crit Care Med 2012; 40: 2654–2661. doi:10.1097/CCM.0b013e3182592021 22743778

[23] Fiamma MN, Straus C, Thibault S, et al. Effects of hypercapnia and hypocapnia on ventilatory variability and the chaotic dynamics of ventilatory flow in humans. Am J Physiol Regul Integr Comp Physiol 2007; 292: R1985–R1993. doi:10.1152/ajpregu.00792.2006 17218438

[24] El-Khatib M, Jamaleddine G, Soubra R, et al. Pattern of spontaneous breathing: potential marker for weaning outcome. Spontaneous breathing pattern and weaning from mechanical ventilation. Intensive Care Med 2001; 27: 52–58. doi:10.1007/s001340000758 11280673

[25] Engoren M. Approximate entropy of respiratory rate and tidal volume during weaning from mechanical ventilation. Crit Care Med 1998; 26: 1817–1823. doi:10.1097/00003246-199811000-00021 9824073

[26] Park JE, Kim TY, Jung YJ, et al. Biosignal-based digital biomarkers for prediction of ventilator weaning success. Int J Environ Res Public Health 2021; 18: 9229. doi:10.3390/ijerph18179229 34501829 PMC8430549

[27] Brack T, Jubran A, Tobin MJ. Dyspnea and decreased variability of breathing in patients with restrictive lung disease. Am J Respir Crit Care Med 2002; 165: 1260–1264. doi:10.1164/rccm.2201018 11991875

[28] Spieth PM, Carvalho AR, Pelosi P, et al. Variable tidal volumes improve lung protective ventilation strategies in experimental lung injury. Am J Respir Crit Care Med 2009; 179: 684–693. doi:10.1164/rccm.200806-975OC 19151194

[29] Carvalho AR, Spieth PM, Güldner A, et al. Distribution of regional lung aeration and perfusion during conventional and noisy pressure support ventilation in experimental lung injury. J Appl Physiol 2011; 110: 1083–1092. doi:10.1152/japplphysiol.00804.2010 21270348

[30] Spieth PM, Carvalho AR, Güldner A, et al. Pressure support improves oxygenation and lung protection compared to pressure-controlled ventilation and is further improved by random variation of pressure support. Crit Care Med 2011; 39: 746–755. doi:10.1097/CCM.0b013e318206bda6 21263322

[31] Gama de Abreu M, Spieth PM, Pelosi P, et al. Noisy pressure support ventilation: a pilot study on a new assisted ventilation mode in experimental lung injury. Crit Care Med 2008; 36: 818–827. doi:10.1097/01.CCM.0000299736.55039.3A 18431269

[32] Arold SP, Suki B, Alencar AM, et al. Variable ventilation induces endogenous surfactant release in normal guinea pigs. Am J Physiol Lung Cell Mol Physiol 2003; 285: L370–L375. doi:10.1152/ajplung.00036.2003 12851212

[33] Schmidt M, Dres M, Raux M, et al. Neurally adjusted ventilatory assist improves patient–ventilator interaction during postextubation prophylactic noninvasive ventilation. Crit Care Med 2012; 40: 1738–1744. doi:10.1097/CCM.0b013e3182451f77 22610179

[34] Suki B, Alencar AM, Sujeer MK, et al. Life-support system benefits from noise. Nature 1998; 393: 127–128. doi:10.1038/30130 9603516

[35] Spieth PM, Güldner A, Huhle R, et al. Short-term effects of noisy pressure support ventilation in patients with acute hypoxemic respiratory failure. Crit Care 2013; 17: R261. doi:10.1186/cc13091 24172538 PMC4056040

[36] Ball L, Sutherasan Y, Fiorito M, et al. Effects of different levels of variability and pressure support ventilation on lung function in patients with mild-moderate acute respiratory distress syndrome. Front Physiol 2021; 12: 725738. doi:10.3389/fphys.2021.725738 34744766 PMC8569865

