# Supplementary material for: Decreased breathing variability is associated with poorer outcome in mechanically ventilated patients
Source: ERJ Open Res. 2023 May 2;9(3):00544-2022. doi: 10.1183/23120541.00544-2022 (PMC10152249; doi:10.1183/23120541.00544-2022)
Supplement: Supplementary file 1 [file 00544-2022.supplement.pdf]

Decreased breathing variability is associated with poorer outcome in mechanically ventilated patients

Camille Rolland-Debord, M.D., PhD., Tymothee Poitou, M.D, Come Bureau, M.D., Isabelle Rivals, Thomas Similowski, M.D., PhD., Alexandre Demoule, M.D., PhD.

Supplementary material

- **Supplemental methods**
- **Table SDC1. Differences of descriptors of breathing variability and complexity between ventilator modes**
- **Table SDC2. Association between descriptors of breathing variability and complexity, and duration of mechanical ventilation**
- **Table SDC3. Association between descriptors of breathing variability and complexity, and 28-days ventilator-free days**
- **Figure SDC1. Correlation between the duration of mechanical ventilation (MV) and descriptors of breathing variability (see online data supplemental figure)**

Supplemental methods

Written informed consent was obtained from the patients, or their surrogates, before being included in the study.

Patients

Patients receiving MV for more than 24 h for acute respiratory failure of respiratory cause (*de novo* hypoxemic respiratory failure, acute cardiogenic pulmonary edema or acute-on-chronic respiratory failure) were eligible when they met the following criteria: ability to sustain PSV for at least 30 min with a total level of inspiratory pressure less than 30 cmH_2O, estimated remaining duration of mechanical ventilation greater than 48 h, level of sedation less than or equal to four on the Ramsay scale, fraction of inspired oxygen less than or equal to 50% with a positive end-expiratory pressure less than or equal to 8 cmH_2O and absence of administration of high-dose vasopressor therapy.

Patient management

As soon as they were included, patients were connected to a Servo-i ventilator (Maquet Critical Care, Sweden) equipped with NAVA mode. The standard nasogastric feeding tube was removed and replaced by an electrical activity of the diaphragm (EAdi) catheter consisting of a 16-Fr gastric tube equipped with electrodes. Patients were then randomly assigned to receive either PSV or NAVA. The pressure support level in the PSV group and the NAVA level were set to obtain a tidal volume of 6 to 8 ml/kg of ideal body weight. NAVA or PSV was continued unless the patients met predefined criteria for switching back to controlled mechanical ventilation or for weaning and subsequent extubation. The investigators were not involved in any clinical decisions.

Data collection

Data analysis

For each patient, the four 20-minute recordings (12, 24, 36 and 48 hours after inclusion) were merged into one single 80-minute recording session on which analyses were performed (Figure 1).

Breath-by-breath variability

Flow and EAdi-derived breathing pattern variables were determined on a breath-by-breath basis using a MATLAB script (Mathworks, Natick, MA, USA). This automatic verification was followed by a visual inspection of the recording by two investigators. Flow-derived breathing pattern variables included tidal volume and respiratory rate. For EAdi, peak EAdi (EAdi-peak) and EAdi-inspiratory neural time were measured. The coefficient of variation (standard deviation divided by the mean) was then calculated (the higher the coefficient of variation, the higher the variability).

Spectral analysis

We assessed spectral-derived variability using the amplitude ratio of the spectrum's first harmonic (H1) to its zero-frequency or DC component (H1/DC) according to the method described by Gutierrez et al. [1] (the higher the H1/DC, the lower the variability). H1/DC was calculated for the flow and the EAdi signal. For inspiratory H1/DC, flow expiratory values were set to zero. For expiratory H1/DC, flow inspiratory values were set to zero. This resulted in a periodic, continuous signal, displaying only either the inspiratory or expiratory phase of the cycle. A frequency spectrum was generated by applying the Cooley-Tukey Fast Fourier Transform algorithm [2] to the modified signal data encompassed by a predetermined time window of constant duration. The length of the time window was mandated by the Fast Fourier Transform requirement of 2n samples per frequency spectrum. In order to fit with Gutierrez's method, we chose 4096 (2^{12}) samples, resulting in a time window of 0.7 min at sampling rate

of 100 Hz. The resulting spectrum had a frequency resolution of 24.41 x 10^{-3} Hz. For each spectrum, the H1/DC was calculated with a peak detection algorithm. The same procedure was applied to the EAdi signal.

Breathing complexity

In humans, ventilatory flow is not a truly periodic phenomenon. Its variability from breath to breath exhibits chaos-like mathematical complexity [3]. This means that the trajectory of ventilatory flow is nonlinear, bounded, and not predictable in the long term. As previously described by our group, we carried out complexity analysis of continuous oscillatory signals (flow and EAdi). Consecutive breaths are described in terms of an ensemble of signal trajectories. If only one trajectory is possible, as in a truly periodic system, complexity is minimal, and all breaths should be identical.

After subsampling the signal at 5 Hertz, the noise titration procedure was performed as described previously [4-6], first ascertains the presence of nonlinearity in the signal through a statistical process, and then it quantifies the amount of added white noise needed to mask this nonlinearity. A noise limit above 0 means nonlinearity and a certain degree of chaos-compatible complexity.

Complex dynamical systems exhibit unpredictable behaviors such that small variances in the initial conditions could have profound and widely divergent effects on the system's outcomes. We quantified the sensitivity to initial conditions for flow and EAdi using the largest Lyapunov exponent (LLE), which increases, as the system is more sensitive to initial conditions [7], which increases, as the system is more sensitive to initial conditions.

Statistics

As this is an ancillary study, no sample size could be calculated to detect a difference. The sample size was determined by the parent study [8]. Statistical analysis was performed with GraphPad (GraphPad Software, San Diego, CA, USA) and R (The R Foundation, Vienna, Austria). Continuous data were reported as median (interquartile range) and categorical data as number of events (percentages). Continuous variables (i.e. duration of MV and number of 28-day VFDs) were dichotomized according to their median value in the population.

Differences between groups were assessed with the Mann-Whitney test for continuous variables and with the Chi-2 test for categorical variables. Each potential risk factor for death was first evaluated in a univariate model. Then, a multivariate logistic regression analysis was performed. The multivariate model was built with variables that yielded p values of less than 0.2 on univariate analysis. The adjusted odds ratio (OR) of variables present in the final model is presented with a 95% confidence interval (CI). Finally, correlation between duration of MV, 28-day VFDs and descriptors of breathing variability were evaluated using Spearman's rank correlation coefficient.

1. Gutierrez G, Ballarino GJ, Turkan H, Abril J, De La Cruz L, Edsall C, George B, Gutierrez S, Jha V, Ahari J. Automatic detection of patient-ventilator asynchrony by spectral analysis of airway flow. *Crit Care* 2011: 15(4): R167.
2. Duhamel P, Vetterli M. Fast Fourier transforms: a tutorial review and a state of the art. Signal Processing (Elsevier), 1990; pp. 259-299.
3. Straus C, Samara Z, Fiamma MN, Bautin N, Ranohavimparany A, Le Coz P, Golmard JL, Darré P, Zelter M, Poon CS, Similowski T. Effects of maturation and acidosis on the chaos-like complexity of the neural respiratory output in the isolated brainstem of the tadpole, Rana esculenta. *Am J Physiol Regul Integr Comp Physiol* 2011: 300(5): R1163-1174.
4. Mangin L, Fiamma MN, Straus C, Derenne JP, Zelter M, Clerici C, Similowski T. Source of human ventilatory chaos: lessons from switching controlled mechanical ventilation to inspiratory pressure support in critically ill patients. *Respir Physiol Neurobiol* 2008: 161(2): 189-196.

5. Schmidt M, Kindler F, Cecchini J, Poitou T, Morawiec E, Persichini R, Similowski T, Demoule A. Neurally adjusted ventilatory assist and proportional assist ventilation both improve patient-ventilator interaction. *Crit Care* 2015: 19: 56.

6. Roulin E, Freitas US, Letellier C. Working conditions for safe detection of nonlinearity and noise titration. *Phys Rev E Stat Nonlin Soft Matter Phys* 2011: 83(4 Pt 2): 046225.

7. Briggs K. An improved method for estimating Liapunov exponents of chaotic time series. Physics Letters A, 1990.

8. Demoule A, Clavel M, Rolland-Debord C, Perbet S, Terzi N, Kouatchet A, Wallet F, Roze H, Vargas F, Guerin C, Dellamonica J, Jaber S, Brochard L, Similowski T. Neurally adjusted ventilatory assist as an alternative to pressure support ventilation in adults: a French multicentre randomized trial. *Intensive Care Med* 2016: 42(11): 1723-1732.

Table SDC1. Differences of descriptors of breathing variability and complexity between ventilator modes

	PSV	NAVA	p
Coefficient of variation			
Tidal volume, *%*	18 (15-25)	28 (19-36)	0.0014
Respiratory rate, *%*	22 (18-30)	23 (18-28)	0.9162
EAdi-peak, *%*	36 (30-52)	34 (25-44)	0.1912
EAdi–inspiratory neural time, *%*	31 (26-39)	32 (27-41)	0.7850
H1/DC			
Inspiratory flow, *%*	37 (31-41)	40 (32-48)	0.2280
Inspiratory EAdi, *%*	39 (33-48)	46 (41-50)	0.0316
Expiratory flow, *%*	23 (18-28)	24 (18-32)	0.4015
Expiratory EAdi, *%*	27 (22-33)	33 (28-38)	0.0015
Complexity			
Noise Limit flow, *%*	46 (34-63)	53 (36-70)	0.2294
Noise Limit EAdi, *%*	46 (34-64)	52 (35-71)	0.2469
LLE flow, *bit.iteration*$^{-1}$	1.7 (1.4-2.3)	2.5 (2.1-3.1)	<0.0001
LLE EAdi, *bit.iteration*$^{-1}$	0.20 (0.1-0.4)	0.21 (0.14-0.33)	0.6570

EAdi, electrical activity of the diaphragm; H1/DC, amplitude ratio of the first harmonic peak (H1) to that of zero frequency or DC component; LLE, largest Lyapunov exponent.

Table SDC2. Association between descriptors of breathing variability and complexity, and duration of mechanical ventilation

	Duration of mechanical ventilation		
	**Spearman**	**IC95%**	**p**
Coefficient of variation			
Tidal volume, *%*	-0.17	[-0.37;0.04]	0.099
Respiratory rate, *%*	-0.06	[-0.28;0.15]	0.542
EAdi-peak, *%*	-0.12	[-0.32;0.09]	0.272
EAdi–inspiratory neural time, *%*	-0.05	[-0.25;0.16]	0.641
H1/DC			
Inspiratory flow, *%*	0.23	[0.02;0.41]	0.030
Inspiratory EAdi, *%*	0.27	[0.07;0.45]	0.008
Expiratory flow, *%*	0.07	[-0.16;0.26]	0.493
Expiratory EAdi, *%*	0.13	[-0.11;0.32]	0.216
Complexity			
Noise Limit flow, *%*	0.25	[0.04;0.43]	0.015
Noise Limit EAdi, *%*	0.25	[0.04;0.42]	0.018
LLE flow, *bit.iteration*$^{-1}$	0.08	[-0.13;0.27]	0.439
LLE EAdi, *bit.iteration*$^{-1}$	0.06	[-0.15;0.26]	0.560

EAdi, electrical activity of the diaphragm; H1/DC, amplitude ratio of the first harmonic peak (H1) to that of zero frequency or DC component; LLE, largest Lyapunov exponent.

Table SDC3. Association between descriptors of breathing variability and complexity, and 28-days ventilator-free days

	28-days ventilator-free days		
	**Spearman**	**IC95%**	**p**
Coefficient of variation			
Tidal volume, *%*	0.22	[0.02;0.41]	0.034
Respiratory rate, *%*	0.19	[-0.03;0.38]	0.076
EAdi-peak, *%*	0.12	[-0.09;0.32]	0.244
EAdi–inspiratory neural time, *%*	0.03	[-0.18;0.24]	0.773
H1/DC			
Inspiratory flow, *%*	-0.43	[-0.58;-0.24]	p<0.0001
Inspiratory EAdi, *%*	-0.46	[-0.61;-0.28]	p<0.0001
Expiratory flow, *%*	-0.24	[-0.41;-0.01]	0.019
Expiratory EAdi, *%*	-0.31	[-0.47;-0.10]	0.002
Complexity			
Noise Limit flow, *%*	-0.32	[-0.49;0.12]	0.001
Noise Limit EAdi, *%*	-0.32	[-0.47;0.10]	0.002
LLE flow, *bit.iteration*$^{-1}$	-0.21	[-0.39;-0.002]	0.042
LLE EAdi, *bit.iteration*$^{-1}$	0.02	[-0.18;0.23]	0.818

EAdi, electrical activity of the diaphragm; H1/DC, amplitude ratio of the first harmonic peak (H1) to that of zero frequency or DC component; LLE, largest Lyapunov exponent.

Figure SDC1. Correlation between the duration of mechanical ventilation (MV) and descriptors of breathing variability (A) coefficient of variation of Tidal volume (%), (B) H1/DC inspiratory flow, *%*, (C) H1/DC inspiratory EAdi, *%*, (D) H1/DC expiratory flow, *%*, (E) H1/DC expiratory EAdi, *%*, (F) Noise Limit flow, *%*, (G) Noise Limit EAdi, *%*, (H) LLE flow, *bit.iteration*$^{-1}$ evaluated using Spearman's rank correlation coefficient.

Abbreviation: EAdi, electrical activity of the diaphragm; H1/DC, amplitude ratio of the first harmonic peak (H1) to that of zero frequency (also termed DC component).

Figure SDC1.

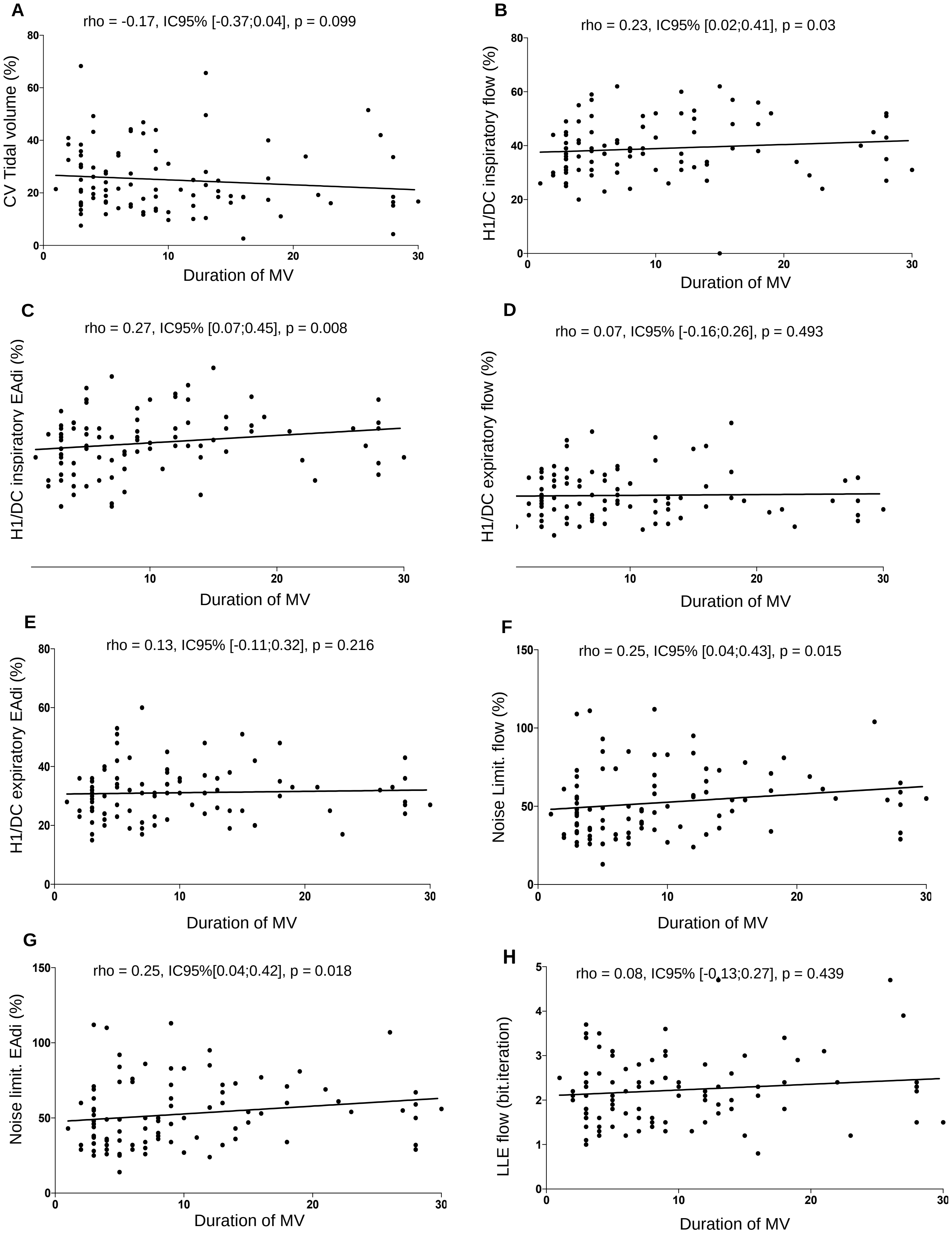
A
rho = -0.17, IC95% [-0.37;0.04], p = 0.099
CV Tidal volume (%)
Duration of MV
B
rho = 0.23, IC95% [0.02;0.41], p = 0.03
H1/DC inspiratory flow (%)
Duration of MV
C
rho = 0.27, IC95% [0.07;0.45], p = 0.008
H1/DC inspiratory EAdi (%)
Duration of MV
D
rho = 0.07, IC95% [-0.16;0.26], p = 0.493
H1/DC expiratory flow (%)
Duration of MV
E
rho = 0.13, IC95% [-0.11;0.32], p = 0.216
H1/DC expiratory EAdi (%)
Duration of MV
F
rho = 0.25, IC95% [0.04;0.43], p = 0.015
Noise Limit. flow (%)
Duration of MV
G
rho = 0.25, IC95%[0.04;0.42], p = 0.018
Noise limit. EAdi (%)
Duration of MV
H
rho = 0.08, IC95% [-0.13;0.27], p = 0.439
LLE flow (bit.iteration)
Duration of MV
